# Platelet-Reactive Antibodies in Patients after Ischaemic Stroke—An Epiphenomenon or a Natural Protective Mechanism

**DOI:** 10.3390/ijms21218398

**Published:** 2020-11-09

**Authors:** Young Eun Park, Rushi Penumarthy, Paul P. Sun, Caroline Y. Kang, Marie-Christine Morel-Kopp, Jonathan Downing, Taryn N. Green, Tracey Immanuel, Christopher M. Ward, Deborah Young, Matthew J. During, P. Alan Barber, Maggie L. Kalev-Zylinska

**Affiliations:** 1 Blood and Cancer Biology Laboratory, Department of Molecular Medicine & Pathology, University of Auckland, Auckland 1142, New Zealand; y.park@auckland.ac.nz (Y.E.P.); rpen032@aucklanduni.ac.nz (R.P.); wanli@ivd-probio.com (P.P.S.); kangyaming@msn.com (C.Y.K.); t.green@auckland.ac.nz (T.N.G.); tracey.immanuel@auckland.ac.nz (T.I.); 2 Department of Haematology and Transfusion Medicine, Royal North Shore Hospital, Sydney 2065, Australia; marie-christine.kopp@sydney.edu.au (M.-C.M.-K.); chris.ward@sydney.edu.au (C.M.W.); 3 Northern Blood Research Centre, Kolling Institute, University of Sydney, Sydney 2065, Australia; 4 New Zealand Blood Service Centre, Auckland 1051, New Zealand; Jonathan.Downing@health.wa.gov.au; 5 Department of Pharmacology and Clinical Pharmacology, University of Auckland, Auckland 1142, New Zealand; ds.young@auckland.ac.nz; 6 Centre for Brain Research, University of Auckland, Auckland 1142, New Zealand; matt@meiragtx.com (M.J.D.); ABarber@adhb.govt.nz (P.A.B.); 7 Departments of Molecular Virology, Immunology and Medical Genetics, Neuroscience and Neurological Surgery, Ohio State University, Columbus, OH 43210, USA; 8 Department of Neurology, Auckland City Hospital, Auckland 1148, New Zealand; 9 Department of Pathology and Laboratory Medicine, LabPlus Haematology, Auckland City Hospital, Auckland 1148, New Zealand

**Keywords:** thrombosis, stroke, autoantibodies, anti-platelet antibodies, platelet inhibition, neuroprotection, protective autoimmunity

## Abstract

Ischaemic brain damage induces autoimmune responses, including the production of autoantibodies with potential neuroprotective effects. Platelets share unexplained similarities with neurons, and the formation of anti-platelet antibodies has been documented in neurological disorders. The aim of this study was to investigate the presence of anti-platelet antibodies in the peripheral blood of patients after ischaemic stroke and determine any clinical correlations. Using a flow cytometry-based platelet immunofluorescence method, we detected platelet-reactive antibodies in 15 of 48 (31%) stroke patients and two of 50 (4%) controls (*p* < 0.001). Western blotting revealed heterogeneous reactivities with platelet proteins, some of which overlapped with brain proteins. Stroke patients who carried anti-platelet antibodies presented with larger infarcts and more severe neurological dysfunction, which manifested as higher scores on the National Institutes of Health Stroke Scale (NIHSS; *p =* 0.009), but they had a greater recovery in the NIHSS by the time of hospital discharge (day 7 ± 2) compared with antibody-negative patients (*p* = 0.043). Antibodies from stroke sera reacted more strongly with activated platelets (*p* = 0.031) and inhibited platelet aggregation by up to 30.1 ± 2.8% (*p <* 0.001), suggesting the potential to interfere with thrombus formation. In conclusion, platelet-reactive antibodies can be found in patients soon after ischaemic stroke and correlate with better short-term outcomes, suggesting a potential novel mechanism limiting thrombosis.

## 1. Introduction

During ischaemic stroke, the blood–brain barrier (BBB) becomes damaged, and intracellular proteins released from the necrotising brain tissue come in contact with the peripheral immune system inducing humoral responses [1]. As a result, stroke patients develop autoantibodies that target intracellular neuronal and glial antigens. These antigens include brain-specific neurofilaments, glial fibrillary acidic protein, S100 calcium-binding protein B (S100B), myelin basic protein, proteolipids, and degradation products of neuronal surface receptors such as *N*-methyl-D-aspartate (NMDA) receptor subunits GluN1, GluN2A, and GluN2B [2,3,4,5,6]. Elevated levels of such autoantibodies, including immunoglobulin G (IgG) class, are detected within hours to days after stroke onset, suggesting that patients encounter the antigen before the stroke [5,6,7]. Yet, very little is known about what triggers antibody formation or what effects these autoantibodies have in stroke patients.

There is evidence to suggest that autoantibodies to platelet antigens may support stroke outcomes. Members of the S100 family of calcium-binding proteins are expressed in multiple cell types, including neurons and platelets [8]. The platelet-derived S100A9 protein is up-regulated on the surface of activated platelets and secreted extracellularly both in vitro and in mice [9]. Platelets isolated from patients with myocardial infarction express higher levels of S100A9 compared with patients with stable coronary artery disease [10]. Studies in knockout mice demonstrated that S100A9 increases thrombus formation through binding to the platelet CD36 receptor [9]. S100A9 also promotes leukocyte recruitment to the site of an atherosclerotic plaque, which drives vascular inflammation [11]. To inhibit the atherothrombotic functions of S100A9, an experimental vaccine was developed targeting the C-terminal protein domain [12]. Vaccinated mice achieved sustainable increase in anti-S100A9 antibody levels and were protected against thrombosis. Brain infarct was restricted in two different models of middle cerebral artery occlusion, and platelet inhibition lasted more than 2 months, with no associated risk of bleeding or adverse autoimmune responses [12]. These results provide a proof of concept that autoantibodies targeting selected platelet antigens contribute to a safe and long-acting platelet inhibition with the potential to improve stroke outcomes.

We hypothesised that platelet-reactive autoantibodies can be found in stroke patients due to antigens that are shared between neurons and platelets. Using a sensitive flow cytometry-based immunofluorescence method, platelet-reactive antibodies were examined in the peripheral blood of patients after stroke. Antibody presence was correlated with clinical and radiological measures of stroke severity in patients, and antibody effects on platelets were tested in vitro using light transmission aggregometry.

## 2. Results

Forty-eight patients with ischaemic stroke (mean age 70 ± 17 years; 25 [52%] women) were tested by flow cytometry for the presence of antibodies targeting human platelets. Characteristics of stroke patients at inclusion into the study are shown in Table 1.

The National Institutes of Health Stroke Scale (NIHSS) score ranges from 0 to 23, where higher scores indicate higher stroke severity and poorer prognosis [13]. The median NIHSS score for patients on admission was 5 (1–23). The Alberta Stroke Program Early CT (computed tomography) scale (ASPECT) assesses the extent of the middle cerebral artery occlusion based on CT appearances; a score of 10 is normal and 0 indicates diffuse ischaemia [14]. Our patients had CT brain scans performed at a median of 3 h and 20 min from hospital admission. The median ASPECT score was 9 (3–10). Cortical involvement was seen in 35% of patients. Cardiac embolism was the most frequent cause of the stroke (38%), and 29% of infarcts were because of small vessel disease. Four patients (8%) received thrombolytic treatment with recombinant tissue–plasminogen activator (rt-PA, Alteplase). The control subjects (*n* = 50 healthy blood donors, mean age 55 ± 6 years; 25 women) had no history of prior stroke or an autoimmune disease. 

### 2.1. Platelet-Reactive Antibodies are Found in Peripheral Blood of Patients after Stroke

**Figure 1:**
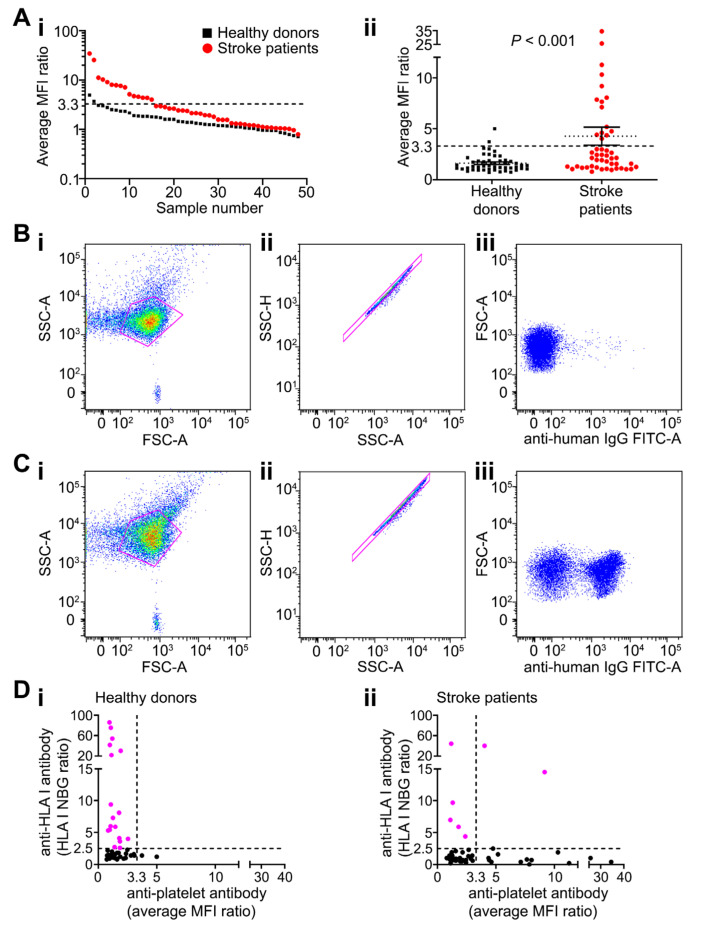
Characterisation of anti-platelet antibodies in patients and controls. (**A**) Anti-platelet antibody levels detected in healthy blood donors and stroke patients are shown in order of decreasing levels of the MFI ratio for individual samples (log scale; (**Ai**)) or in a scatterplot with mean ± SEM of the average MFI ratio (linear scale; (**Aii**)). Data points are mean of three replicates; *n* = 48 of stroke patient and 50 healthy blood donors. *p*-values are shown (Mann–Whitney U test). Dashed lines represent mean + 2 SD of controls (y = 3.3). MFI ratio was calculated as MFI of test subject divided by MFI of negative control. (**B**,**C**) Representative dot plot examples of anti-platelet antibodies in sera from anti-platelet antibody negative and positive patients respectively. (**Bi**,**Ci**) Dot plots of FSC-A versus SSC-A of all events with platelets gated. (**Bii**,**Cii**) Dot plots of SSC-A versus SSC-H excluding doublets within a single cell gate. Examples of negative (**Biii**) and positive (**Ciii**) detection of anti-platelet antibodies in stroke sera generated by a secondary anti-human IgG antibody conjugated to FITC. (**D**) Relationship between anti-platelet antibodies and anti-HLA class I antibodies in healthy blood donors (**Di**) and stroke patients (**Dii**). Dashed lines represent positivity thresholds. The threshold for anti-HLA I antibodies was set as the normalised background ratio (NBG) greater than 2.5 on any of the 12 possible HLA I beads (Luminex). The threshold for anti-platelet antibodies was set based on the mean + 2 SD of healthy blood donors (x = 3.3, as shown in A). Data points are mean of three replicates. Pink dots indicate positive samples; *n* = 48 of each group. Abbreviations: FITC, fluorescein isothiocyanate; FSC-A, forward scatter area; IgG, immunoglobulin G; HLA, human leukocyte antigen; MFI, mean fluorescence intensity; NBG, normalised background ratio; SSC-A, side scatter area; SSC-H, side scatter height.

All 48 patients were tested for the presence of anti-platelet antibodies on day 2 ± 1 after stroke using platelets from three different blood donors of blood group O. Fifteen patients (31%) had anti-platelet antibodies detected by flow cytometry, compared with only two of 50 (4%) blood donors (Figure 1A–C, Supplementary Figure S1). The mean antibody levels (reflected by the average of the mean fluorescence intensity [MFI] from three independent platelet preparations) were higher in stroke patients compared with control subjects (4.3 ± 0.9 and 1.6 ± 0.1 respectively; *p* = 0.0009 [Mann–Whitney U-test]; Figure 1Aii).

Seven of 48 stroke patients were positive for antibodies targeting class I human leukocyte antigens (HLA), but levels did not correlate with anti-platelet antibodies (*p* = 0.380; Figure 1Dii). Anti-HLA class I antibodies were more frequent in blood donors; 18 of 50 healthy blood donors had anti-HLA class I antibodies detected but no associated platelet reactivities (Figure 1Di). Only two of 48 stroke patients and seven of 50 healthy donors were positive for anti-HLA class II antibodies (Supplementary Figure S2). Overall, there was no evidence that anti-HLA antibodies accounted for platelet binding detected in patients’ sera.

### 2.2. Patients with Anti-Platelet Antibodies Had Larger Strokes but Had Better Recovery from Neurological Dysfunction by Day 7 Compared with Antibody-Negative Patients

**Figure 2:**
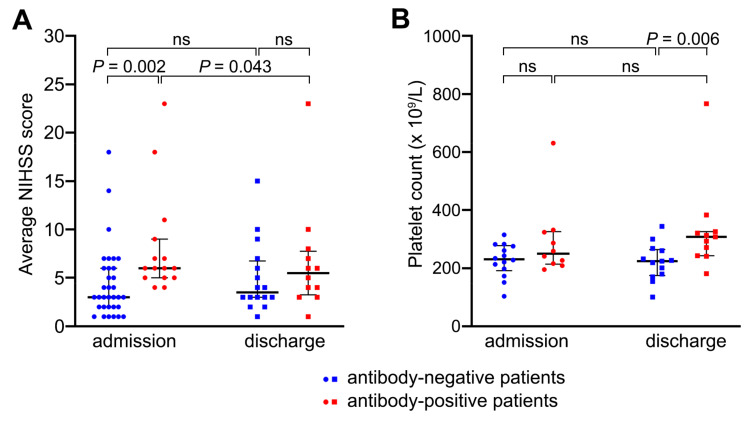
Relationship between the presence or absence of anti-platelet antibodies and patients’ NIHSS scores or platelet counts. Scatterplots plots showing individual NIHSS scores (**A**) and platelet counts (**B**) for antibody-negative patients (blue) and antibody-positive patients (red) recorded at the time of hospital admission (day 0) and discharge (day 7 ± 2). In A, *n* = 16–33 for antibody-negative patients, 12–15 for antibody-positive patients. In B, *n* = 13–14 for antibody-negative patients, 10–11 for antibody-positive patients. Median, interquartile range and *p* values are shown (Wilcoxon test for paired data; Mann–Whitney U test for unpaired data); ns, non-significant.

There was no difference in age, gender, and risk factor profile between stroke patients with or without anti-platelet antibodies (Table 1). However, patients with anti-platelet antibodies more often had total anterior circulation infarcts (TACI) associated with higher NIHSS scores on admission, indicating a more severe neurological deficit on presentation (median NIHSS score of 6, range 4–23) than negative patients (median NIHSS score of 3, range 1–18; *p* = 0.002; Table 1; Figure 2A). The levels of anti-platelet antibodies (MFI values) positively correlated with the admission NIHSS scores (linear regression, R^2^ = 0.248, *p* < 0.001) and at discharge (R^2^ = 0.376, *p* = 0.001; Supplementary Table S1). Congruently, there was a negative correlation between anti-platelet antibody levels and the ASPECT score, where a higher score means fewer areas of the brain with infarction (R^2^ = 0.176, *p* = 0.003; Supplementary Table S1). Overall, findings implied that patients with larger strokes were more likely to have higher levels of anti-platelet antibodies.

However, the relationship between the level of anti-platelet antibodies and the NIHSS score weakened by the time of hospital discharge 7 ± 2 days after stroke onset (Figure 2A, Table 2). At discharge, the NIHSS scores declined the most in anti-platelet antibody-positive patients (*p* = 0.043) and mildly increased in antibody-negative patients (Figure 2A; Table 1 and Table 2), supporting better short-term improvement in patients displaying anti-platelet antibodies.

### 2.3. Anti-Platelet Antibodies Did Not Cause Platelet Consumption

Platelet counts on admission were similar between antibody-positive and antibody-negative patients (292 ± 18 × 10^9^/L and 227 ± 8 × 10^9^/L respectively; *p* = 0.106), which were both well within the normal range (150–400 × 10^9^/L). However, by the time of discharge, patients with anti-platelet antibodies had higher platelet counts than negative patients (332 ± 22 × 10^9^/L and 222 ± 9 × 10^9^ /L respectively; *p* = 0.028; Figure 2B), suggesting a stronger reactive response in positive patients. There was a positive correlation between the level of anti-platelet antibodies and platelet counts, which applied to the counts measured on admission (R^2^ = 0.565, *p* < 0.001) and discharge (R^2^ = 0.341, *p* = 0.003; Supplementary Table S1). Higher platelet counts in antibody-positive patients argued against any significant consumption of circulating platelets by the antibodies detected.

### 2.4. Anti-Platelet Antibody Targets are Heterogenous in Stroke Patients But Often Overlap with Brain Proteins

**Figure 3:**
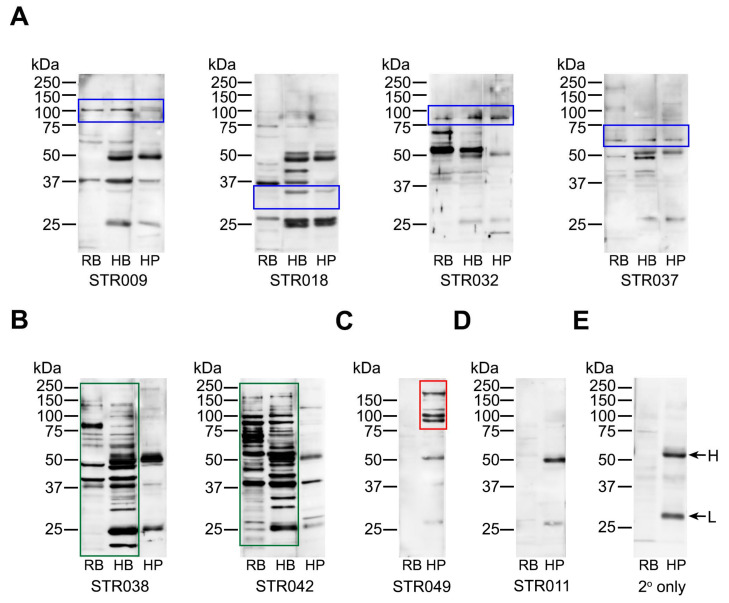
Western blots showing binding patterns of stroke-derived antibodies with brain and platelet proteins. Stroke sera diluted 1:500 were tested for binding to proteins extracted from rat brain tissue (RB), human brain tissue (HB), and human platelets (HP). (**A**) Examples of antibodies binding to platelets by flow cytometry and showing overlapping reactivities with brain proteins (blue frames). (**B**) Examples of antibodies reacting strongly with brain tissue (green frames) but negative for binding to platelets by flow cytometry. The common band at 37 kDa position was non-discriminatory and thus considered non-specific. (**C**) Examples of antibodies not reacting with brain tissue but reacting with platelets (including by flow cytometry; red frame). (**D**) Examples of antibodies negative by Western blotting for both brain and platelet proteins but positive for platelet binding by flow cytometry. (**E**) Binding pattern generated by the secondary antibody only (anti-human IgG diluted 1:10,000). The H-labelled arrow points to the heavy chain of human IgG, and the L-labelled arrow points to the light chain of human IgG (inherently present in human serum and brain but not rat brain tissue). STR codes refer to the individual samples from stroke patients. All original Western blot pictures are shown in Supplementary Figure S3.

We hypothesised that the platelet-reactive antibodies detected in the blood of stroke patients arose due to the overlap in certain antigens between the brain tissue and platelets. To examine this possibility, stroke sera were tested for binding to brain and platelet proteins using Western blotting (Figure 3, Supplementary Figure S3). Rat brain hippocampus and human brain visual cortex were used as the source of brain antigens in parallel with human platelet proteins, and binding profiles were compared.

The reactivities displayed by stroke-derived antibodies were heterogeneous; each patient had antibodies that reacted differently on Western blots, with examples shown in Figure 3 and Supplementary Figure S3. Nevertheless, overlapping bands were seen between neuronal and platelet proteins in some patients (Figure 3A, blue frames), suggesting that common epitopes accounted for the platelet reactivities detected in these samples. However, this did not apply to all, with some patients having antibodies that reacted strongly with brain tissue (Figure 3B, green frames) but not with platelets. There were also patients whose antibodies reacted with platelets but not with brain tissue (Figure 3C, red frame), some of which were only detected by flow cytometry (Figure 3D). The latter findings raised the possibility that platelet antigens, including those that are activation-dependent, triggered antibody formation in some patients.

### 2.5. Anti-Platelet Antibodies Found in Stroke Patients Bind More Strongly to Activated Platelets and Inhibit Platelet Aggregation

**Figure 4:**
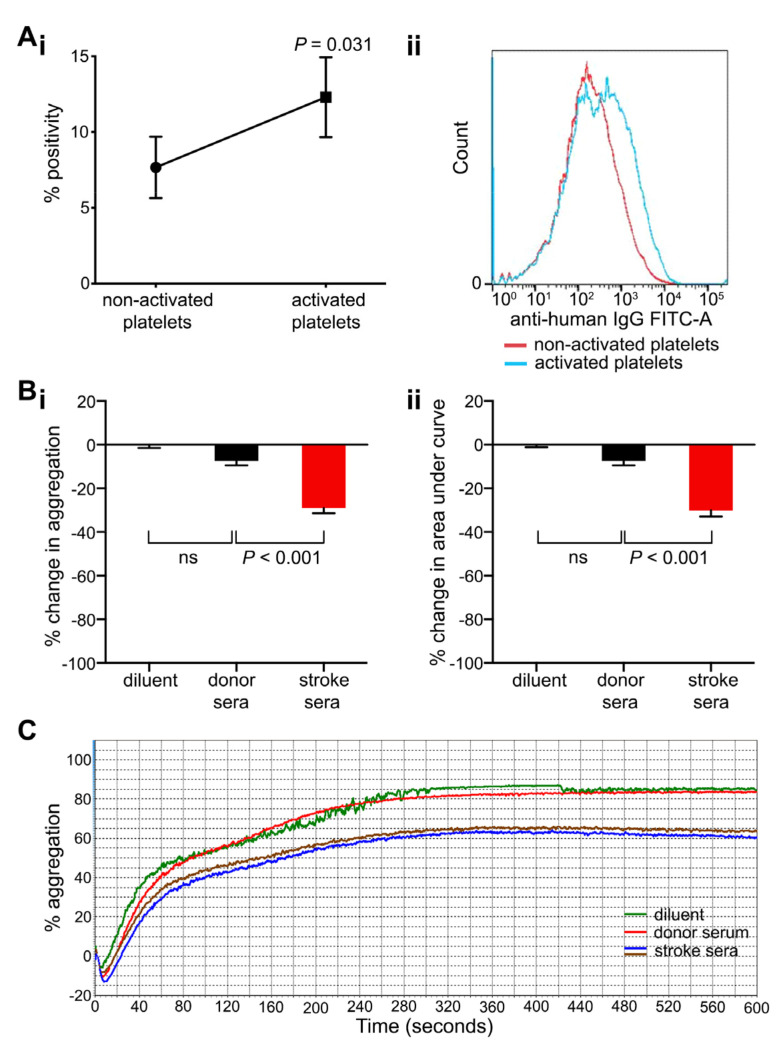
Binding of stroke-derived antibodies to adenosine diphosphate (ADP)-activated platelets and impact of sera on ADP-induced platelet aggregation. (**A**) Antibody binding to platelets tested without and with ADP (i.e., resting and activated platelets), graphed as percentage positivity (percentage of platelets in a positive gate compared to negative control; (**Ai**). Sera were collected from patients at the time of discharge. Data points are mean ± SEM; *n* = 6; *P* values are shown (Wilcoxon test). (**Aii**) Representative histogram example showing an increased fluorescence of anti-platelet antibody binding (generated by a secondary anti-human IgG antibody conjugated to FITC) on platelets activated with 2.5 μM ADP (blue line) compared to non-activated platelets treated with 100 μM aspirin (red line). (**B**) Relative changes in aggregation induced by ADP (2.5–10 μM) seen in the presence of stroke and control sera (100 μL) graphed as percentage change in maximum aggregation (**Bi**) and area under the curve (**Bii**). Data points are mean ± SEM; *n* = 5 (donor sera), 10 (stroke sera); *p*-values are shown (one-way ANOVA with Scheffé post hoc); ns, non-significant. (**C**) Representative example of aggregation traces induced by 5 μM ADP in the presence of stroke and control sera.

To examine if platelet activation could play a role in antibody binding, flow cytometry-based platelet immunofluorescence testing was repeated for selected stroke sera using activated and non-activated platelets. Freshly collected, healthy donor-derived platelets (platelet-rich plasma, PRP) were activated with 2.5 μM adenosine diphosphate (ADP) or platelet resting state was preserved using 100 μM aspirin, and stroke sera were added (*n* = 6) to examine antibody binding. Stroke sera used in these experiments were collected at the time of hospital discharge from patients with a positive anti-platelet antibody screen on admission. In this setting, the binding of stroke antibodies was higher to ADP-activated platelets than resting platelets (mean percentage positivity 12.3 ± 2.6% and 7.7 ± 2.0% respectively; *p* = 0.031; Figure 4A).

Light transmission aggregometry was employed to examine if anti-platelet antibodies from stroke patients had the potential to impact platelet function (Figure 4B,C). ADP-induced platelet aggregation (2.5–10 μM) of donor-derived PRP was measured after pre-incubation with either stroke or control sera (10–100 μL). Antibody-positive sera caused a stronger inhibition of platelet aggregation than control sera. Maximum aggregation and area under the curve were significantly reduced in the presence of stroke sera by 29.0 ± 2.4% and 30.1 ± 2.8% respectively compared with control sera of healthy blood donors (7.4 ± 2.4% each; Figure 4B,C; *p* < 0.001).

## 3. Discussion

After stroke, anti-platelet antibodies are found in patients’ blood, and their presence correlates with better short-term outcomes. We found that antibody-positive patients presented with larger infarcts and more severe neurological dysfunction, supporting the notion that damaged brain tissue provides a trigger for antibody formation. However, by the time of hospital discharge, antibody-positive patients had stronger decline in the NIHSS scores than antibody-negative patients. In vitro, stroke-derived anti-platelet antibodies showed increased reactivity towards activated platelets and inhibited ADP-induced platelet aggregation, suggesting potential protective effect against arterial thrombosis.

Stroke-induced ischaemic cell damage leads to the release of molecules through the injured BBB that activate the peripheral immune system [15,16]. The influx of lymphocytes to the damaged area and the exposure of brain antigens to the immune system can lead to neuroinflammation and the initiation of autoimmunity [15]. Such autoimmunity can be deleterious or protective, depending on lymphocyte type and timing of activation [15]. Pro-inflammatory T cells including CD4-positive, CD8-positive, and γδ-T cells have a deleterious role after stroke. When these cells are depleted, infarct volume is reduced, and functional outcome is improved [17]. In contrast, B cells have been more commonly associated with protective effects after stroke. The beneficial effects observed in mouse models include reduced infarct size, increased post-stroke neurogenesis, and better recovery of motor function [18,19,20,21]. Monoclonal antibodies targeting signalling pathways activated after stroke (e.g., certain inflammatory molecules and ion channels) are being evaluated as potential therapies in pre-clinical stroke models [22]. Nevertheless, there are also studies suggesting that B cells have no impact or are detrimental after stroke [23,24,25]. Thus, the immune response to stroke is a multifaceted process, and there is evidence of both detrimental and beneficial effects.

In this study, detrimental effects cannot be excluded as patients with anti-platelet antibodies presented with larger strokes and higher baseline NIHSS scores, indicating greater neurological impairment. However, by the time of discharge from the hospital, antibody-positive patients had recovered their neurological dysfunction better than antibody-negative patients. Possible mechanisms for the improved recovery include an inhibition of thrombus formation or better thrombus dissolution. In support of such possibilities, sera from stroke patients displayed stronger binding to activated platelets and a stronger inhibition of platelet aggregation in vitro, suggesting potential to interfere with thrombus formation in vivo.

It is unclear what the mechanism of anti-platelet antibody production was in stroke patients. The correlation between anti-neuronal and anti-platelet antibodies observed suggests that the release of necrotising neuronal antigens (through the transiently damaged BBB) provided a trigger for antibody formation, which then led to platelet binding if common epitopes were present. However, for patients whose sera showed no binding to brain proteins, the trigger may have been linked with thrombus formation. Activated platelets are known to up-regulate the expression of a variety of epitopes on their surface, which may induce antibody formation. When platelets are activated, they also increase their surface area, which could allow more physical contact with the antibodies [26].

Patients who were positive for anti-platelet antibodies showed higher platelet counts both at admission and discharge from hospital compared to antibody-negative patients. It is possible that the association between the presence of the antibodies and higher platelet counts is circumstantial, as platelet production can respond to a number of reactive triggers, including inflammation. Greater infarct volume increases the risk of infection, which could also increase the platelet count [27,28]. Conversely, antibody-mediated platelet destruction leads to low platelet counts in patients with immune thrombocytopenic purpura (ITP) [29]. Anti-platelet antibodies are detected in approximately 60% of ITP patients, and they predominantly target platelet glycoprotein (GP) IIb/IIIa and/or the GP Ib–IX–V complex [29]. Antibody-mediated platelet clearance occurs via splenic macrophages and dendritic cells, complement deposition, and platelet apoptosis but antibody-mediated inhibition of platelet production by bone marrow megakaryocytes may also contribute to thrombocytopenia in ITP [30,31,32,33]. As stroke patients had normal platelet counts, our findings suggest that platelet-reactive antibodies did not cause significant platelet consumption nor target the same antigens as the antibodies found in ITP, which points towards a different mechanism operating in stroke patients.

One of the most common forms of secondary stroke prevention is the inhibition of platelet activation and aggregation by anti-platelet medication [34,35]. Not all patients will completely respond to treatment, and preliminary evidence suggests that high platelet reactivity, despite treatment, is associated with adverse outcomes after stroke [36,37]. We saw no evidence that during the period of hospitalisation, patients with anti-platelet antibodies had increased rates of bleeding complications (data not shown). However, further assessment of bleeding risk while on anti-platelet drugs outside of this time period would be an important outcome for future studies in this area. Recently, a novel monoclonal antibody fragment targeting platelet glycoprotein VI (GPVI) caused an inhibition of platelet aggregation in healthy human subjects with no change in the platelet count [38]. A similar approach offers neuroprotection in a mouse model of stroke with no increase in bleeding complications [39]. Our Western blots provided no evidence that stroke-associated anti-platelet antibodies cross-reacted with GPVI, but these antibodies preferentially bound activated platelets and inhibited platelet aggregation. The observation that platelet counts were unaffected in our patients does not exclude antibody functionality in vivo and may argue in favour of a potential protective effect. Platelet reactivity is often reduced in patients after stroke [40,41]. It may be of interest to examine if this also correlates with outcomes or the presence of anti-platelet antibodies.

IgG autoantibodies were detected in patients’ blood within 48 h of stroke onset, which suggests that patients’ immune systems had encountered the antigenic trigger before. Indeed, previous stroke was more common in antibody-positive patients (six of 15 [40%] had previous stroke) than in antibody-negative patients (eight of 33; 24%), which may have provided an immune trigger, but the difference was not statistically significant (*p* = 0.266). The sample size was small, so this warrants further testing. We documented an established vascular risk factor in 14 of 15 (93%) anti-platelet positive patients. Most patients had two risk factors, but this was not significantly different from anti-platelet negative patients. The presence of anti-platelet antibodies had no significant link to any single measured risk factor (Table 1). Nevertheless, it remains more likely that antecedent thrombosis, including possible subclinical brain ischaemia (see below) and neuronal damage primed the immune system to produce the autoantibodies that were detected. In support, anti-GluN2A/B antibody levels are higher in patients with multiple recent vascular events compared to an isolated event [42], and higher levels of anti-NMDA receptor antibodies predict for subsequent neurological events [43].

We were unable to account for an effect of silent brain infarction, which has a prevalence of one in five stroke-free adults [44,45]. It is possible that in the absence of previous stroke or transient ischaemic attack (TIA), anti-platelet antibody-positive patients had a silent brain infarction that allowed exposure to a thrombotic event to occur, triggering an immune reaction. The prevalence of silent brain infarction increases with age, and the risk rises in association with disorders such as hypertension and atrial fibrillation [46,47]. In our cohort, antibody-positive patients tended to be older (*p* = 0.193), and their most common vascular risk factors were hypertension and atrial fibrillation. Hypertension was not differentially distributed between antibody-positive and negative patients, but atrial fibrillation was more common in antibody-positive patients (six of 15; 40%) compared with antibody-negative patients (eight of 33; 24%; *p* = 0.266). We hypothesise that anti-platelet antibodies may arise from a combined number of possible vascular events that lead to thrombosis and trigger an immune reaction, but future studies are required to determine the most relevant risk associations.

This study has a number of limitations. The sample size of 48 stroke patients was relatively small, and out of those, only 15 had anti-platelet antibodies detected. There was no follow-up of patients beyond hospital discharge. Therefore, our findings are preliminary and should be viewed as hypothesis-generating. Although not obvious, we cannot exclude the possibility that a “ceiling effect” in antibody-negative patients influenced the relationship between anti-platelet antibodies and neurological disability. No specific or primary platelet antigenic target was identified, nor is it known what triggered the antibody production. Future studies are required to identify which vascular events correlate with antibody production. It is also unknown what the mechanism of antibody action was in relationship to stroke severity and recovery of neurological deficit. Ideally, we should have tested patients’ own platelets reactivity to ADP directly, but this would have to be done before the implementation of any anti-platelet treatment, which can be difficult to achieve in this group of patients. By using healthy control platelets and comparing effects of antibody-positive stroke sera with antibody-negative sera, a significant decrease in platelet aggregation of 30% on average was observed. In our laboratory, the cutoff level for the normal aggregation response is >50% for 2.5 µM ADP and >75% for 5 and 10 µM ADP, so the average result fell below the reference cutoff. Further characterisation of antibody effects on platelet function is needed. It is also important to exclude any association between anti-platelet antibodies and bleeding complications in patients treated with anti-platelet drugs. To address these limitations, larger sample size is required, which may be more efficiently achieved through a collaborative, multi-centre approach. If our findings are confirmed, the nature of endogenous anti-platelet antibodies may suggest novel and safer ways to inhibit arterial thrombosis.

In summary, platelet-reactive antibodies were detected in one-third (15 of 48) of stroke patients in this study. Patients who carried these antibodies were more likely to have larger infarcts with more severe neurological deficit on admission but recovered their neurological functions better upon discharge than antibody-negative patients. Stroke sera inhibited platelet aggregation, raising the possibility that anti-platelet antibodies may offer a protective function after stroke, which aligns with earlier work showing poorer outcomes in patients with higher platelet reactivity post stroke despite treatment. Future larger-scale studies are warranted to examine the validity of our hypothesis that anti-platelet autoantibodies, by inhibiting platelet function, offer a potential protective effect limiting thrombosis in the acute phase after stroke.

## 4. Materials and Methods

### 4.1. Patients and Controls

This study examined 48 patients with acute ischaemic stroke managed in a specialised stroke unit of a tertiary academic hospital providing care for approximately 1 million people. Initially, 50 patients were recruited, but two were subsequently excluded as found not to have a stroke. Written informed consent was obtained from all participants prior to testing. All study procedures were approved by the Northern X Health and Disability Ethics Committee (approval number AKX/03/07/183 granted on 15 August 2003) and from the Research Development Office of the Auckland District Health Board (approval number 2003/183 A + 2757 granted on 27 July 2004). The same cohort of patients and controls was examined in our previous study for the presence of antibodies targeting the breakdown products of the GluN1 subunit of NMDA receptor [6].

Neurological dysfunction was assessed in patients using the NIHSS score [13]. The score assesses 11 abilities with a number between 0 and 4, 0 being normal functioning and 4 being completely impaired. Some elements only have a scale from 0 to 2, and 42 is the highest total score possible; the higher the number, the more impaired the function. Patients were also classified according to the Trial of Org 10,172 in Acute Stroke Treatment and the Oxfordshire Community Stroke Project (OCSP) clinical criteria [48]. This approach divides patients into total or partial circulation infarcts (TACI or PACI respectively), lacunar infarcts (LACI), and posterior circulation infarcts (POCI). LACI are generally small and subcortical while TACI are large and involve cortical regions. All 48 patients had CT brain scans performed, and 11 patients also had magnetic resonance imaging (MRI) scans. Infarct size and location were classified using the ASPECT score that has a range from 0 to 10, with lower scores indicating larger infarcts [14].

Patients were admitted and had 10 mL of blood collected into BD Vacutainer SST II tubes (BD Biosciences, Franklin Lakes, NJ, USA) for anti-platelet antibody testing within 48 h from stroke onset. Within hours from collection, blood was spun at 4000 *g* for 10 min at 4 °C. Serum was separated and stored in small aliquots at −80 °C until testing. The control group consisted of 50 healthy blood donors recruited from the New Zealand Blood Service Centre. Controls were matched for gender but not perfectly for age, as older people are less likely to donate blood. Donors with history of stroke or autoimmune disease were excluded.

### 4.2. Testing for Anti-Platelet Antibodies

Detection of anti-platelet antibodies was performed using flow cytometry-based platelet immunofluorescence protocol as previously described [49] with minor modifications. Platelets were prepared from the peripheral blood of healthy blood donors collected into Vacutainer tubes containing citrate, theophylline, adenosine, and dipyridamole (CTAD; BD Biosciences, Franklin Lakes, NJ, USA). Platelet donors were of blood group O to exclude the contribution of anti-A and anti-B antibodies to platelet binding. PRP was obtained, and platelets were washed as previously described [50]. Platelets were resuspended in phosphate-buffered saline containing 3 mM dipotassium ethylene diamine tetra-acetic acid (K_2_EDTA), 0.2% (weight per volume; *w*/*v*) bovine serum albumin, and 0.02% (*w*/*v*) sodium azide, at a final concentration of 100–150 × 10^9^/L, and stored at 4 °C before use for a maximum of 2 weeks.

Test sera (10 µL) were incubated with platelets (10 µL) for 30 min at room temperature, followed by a secondary fluorescein-labelled goat-anti-human antibody for 15 min (anti-human IgG-fluorescein isothiocyanate (FITC); 20 µL, diluted 1:100; Jackson Immuno Research, West Grove, PA, USA). Data were acquired on an LSR II flow cytometer (BD Biosciences) as before [50]. The following controls were included in each run: serum known to have anti-platelet antibodies and normal serum (both supplied by the New Zealand Blood Service Centre), secondary antibody only (no human sera), and unstained platelets. Each serum was tested against three platelet preparations obtained from different blood donors, and mean MFI values were derived to reflect anti-platelet reactivity. The test serum was deemed positive if the MFI mean of three anti-IgG-FITC levels exceeded the MFI mean +2 standard deviation (SD) generated by negative control sera.

### 4.3. Platelet Activation

To examine if platelet activation had a bearing on antibody binding, normal human PRP (obtained from healthy donors group O, citrate blood) were prepared within 2 h from blood collection, and antibody binding was re-tested in the presence of 2.5 μM ADP (Helena Laboratories, Beaumont, TX, USA) to induce platelet activation, or with 100 μM acetylsalicylic acid (aspirin; Sigma-Aldrich, Saint Louis, MO, USA) as a negative control [51]. These experiments were conducted using sera samples obtained from stroke patients at the time of discharge from the hospital at 5-9 days after admission.

### 4.4. HLA Antibody Testing

To exclude the contribution to platelet binding from antibodies targeting HLA class I and II molecules, levels of anti-HLA antibodies were measured using a Luminex multiplex assay (Thermo Fisher Scientific, Waltham, MA, USA). The level of HLA antibodies was calculated as a ratio to the negative control (normalised background ratio) with the positivity cutoff set >2.5 of any of the 12 possible HLA class I beads or 5 HLA class II beads [52].

### 4.5. Western Blotting

PRP was prepared and washed as above. Platelet pellets were resuspended in lysis buffer containing 50 mM Tris-HCl (pH 7.5), 2 mM K_2_EDTA, 0.05% Triton X-100, and protease inhibitors (Sigma (St. Louis, MI, USA)). The suspension was incubated on ice for 30 min and vortexed every 5 min. Rat brain lysates (from the excised hippocampus) and human brain lysates (from the visual cortex) were prepared by tissue homogenisation in the lysis buffer (above). Twenty g proteins were separated on 15% sodium dodecyl sulfate polyacrylamide gel electrophoresis (SDS-PAGE) gels, transferred to a nitrocellulose membrane (Hybond-ECL, Amersham, Piscataway, NJ, USA), and processed as previously reported [6]. Membranes were first incubated with diluted human sera (1:500) followed by secondary horseradish-peroxidase–linked anti-human antibodies (1:20,000; Jackson Laboratories). Signals were developed using ECL Plus substrate (Thermo Fisher Scientific, Waltham, MA, USA) in a FujiFilm LAS-3000 phosphoimager (Life Science, Stamford, CT, USA).

### 4.6. Light Transmission Aggregometry

Citrate-PRP and platelet-poor plasma were prepared and platelet aggregation performed using light transmission aggregometry as before [50]. Sera (10–100 µL) from healthy donors and stroke patients were screened for their effects on platelet aggregation. Normal saline (0.9% *w*/*v* NaCl) was used as the additional control. Donor PRP (200 × 10^9^/L platelets) was incubated with patient serum for 1 min at 37 °C prior to ADP stimulation (2.5–10 μM; the lowest ADP concentrations that induced maximum aggregation were used for each platelet donor).

### 4.7. Statistical Analysis

Data are presented as the mean ± standard error of the mean (SEM) or SD, or median (range), as indicated. Pearson χ^2^ test (or Fisher exact test if *n* < 5) were used to examine the statistical difference for data in categories. Mean differences between groups were analysed using independent samples *t*-test (2-sided), Wilcoxon test for paired data, one-way analysis of variance (ANOVA) for parametric data, or Mann–Whitney U test for non-parametric data. General linear model and linear regression were applied to test the relationship between anti-platelet antibody levels and other clinical, radiological, and laboratory variables. Statistical tests were performed using SPSS version 18.0 (Chicago, IL, USA) and GraphPad Prism version 8.4.3 (La Jolla, CA, USA) software. *p*-values < 0.05 were considered statistically significant.

## Figures and Tables

**Table 1:** Clinical characteristics and features of stroke in all patients with stroke, and according to the presence or absence of anti-platelet antibodies.

Variables	All Stroke Patients (*n* = 48)	Anti-Platelet Antibodies		*p*-Value
		Present (*n* = 15)	Absent (*n* = 33)	
Age, mean ± SD in years	70 ± 17	74 ± 12	67 ± 18	0.193 ‡
Females, *n* (%)	25 (52)	9 (60)	16 (48)	0.459 *
Previous stroke, *n* (%)	14 (29)	6 (40)	8 (24)	0.266 *
Previous TIA, *n* (%)	6 (13)	2 (13)	4 (12)	1.000 ^†^
Risk factors, *n* (%)				
Hypertension	33 (69)	11 (73)	22 (67)	0.644 *
Atrial fibrillation	14 (29)	6 (40)	8 (24)	0.266 *
Ischaemic heart disease	12 (25)	3 (20)	9 (27)	0.728 †
Hyperlipidaemia	11 (23)	4 (27)	7 (21)	0.720 †
Current smoker	11 (23)	1 (7)	10 (30)	0.136 †
Peripheral vascular disease	6 (13)	3 (20)	3 (9)	0.360 †
NIHSS score, median (range)				
On admission (day 0)	5 (1–23)	6 (4–23)	3 (1–18)	0.002 §
At discharge (day 7 ± 2)	4 (1–23)	6 (1–23)	4 (1–15)	0.291 §
OCSP, *n* (%)				0.022 *
TACI	5 (10)	4 (27)	1 (3)	0.028 †
PACI	21 (44)	8 (53)	13 (39)	0.367 *
LACI	15 (31)	2 (13)	13 (9)	0.098 †
POCI	7 (15)	1 (7)	6 (18)	0.409 †
ASPECT score, median (range)	9 (3–10)	9 (5–10)	10 (3–10)	0.074 §

Abbreviations: ASPECT, Alberta Stroke Program Early Computed Tomography; LACI, lacunar infarct; NIHSS, National Institutes of Health Stroke Scale; OCSP, Oxfordshire Community Stroke Project; PACI, partial anterior circulation infarct; POCI, posterior circulation infarct; TACI, total anterior circulation infarct; TIA, transient ischaemic attack.* Pearson χ^2^ test; † Fisher’s exact test (2-sided); ‡ Student *t*-test; § Mann–Whitney U test.

**Table 2:** General linear model examining the correlation between anti-platelet antibody levels and the NIHSS score on hospital admission and discharge.

Anti-Platelet Antibody Levels	General Linear Model		
	β-Coefficient Estimate	95% Confidence Interval	*p*-Value
NIHSS score on admission (day 0)	−1.280	−2.480−(−0.080)	0.040
NIHSS score at discharge (day 7 ± 2)	1.185	0.003–2.367	0.050

## Supplementary Materials

The following are available online at https://www.mdpi.com/1422-0067/21/21/8398/s1, Figure S1: Representative examples of controls used for the detection of anti-platelet antibodies by flow cytometry, Figure S2: Comparison between anti-platelet and anti-HLA class II antibodies in patients and controls, Figure S3: Original Western blots showing binding patterns of selected stroke-derived antibodies with brain and platelet proteins. Table S1: Linear regression analysis between the levels of anti-platelet antibodies and the NIHSS score, ASPECT score, and platelet counts in patients after stroke.

## Abbreviations

ADP	adenosine diphosphate
ASPECT	Alberta Stroke Program Early Computed Tomography
BBB	blood–brain barrier
FITC	fluorescein isothiocyanate
FSC-A	forward scatter area
GP	glycoprotein
HLA	human leukocyte antigens
IgG	immunoglobulin G
ITP	immune thrombocytopenic purpura
LACI	lacunar infarct
MFI	mean fluorescence intensity
NBG	normalised background ratio
NIHSS	National Institutes of Health Stroke Scale
NMDA	N-methyl-D-aspartate
OCSP	Oxfordshire Community Stroke Project
PACI	partial anterior circulation infarct
POCI	posterior circulation infarct
PRP	platelet rich plasma
SD	standard deviation
SEM	standard error of the mean
TACI	total anterior circulation infarct
TIA	transient ischaemic attack
SSC-A	side scatter area
SSC-H	side scatter height
w/v	weight per volume
