# Supplementary material for: Platelet-Reactive Antibodies in Patients after Ischaemic Stroke—An Epiphenomenon or a Natural Protective Mechanism
Source: Int J Mol Sci. 2020 Nov 9;21(21):8398. doi: 10.3390/ijms21218398 (PMC7664941; doi:10.3390/ijms21218398)
Supplement: Supplementary file 1 [file ijms-21-08398-s001.pdf]

Appendix A

Supplementary Figure S1.

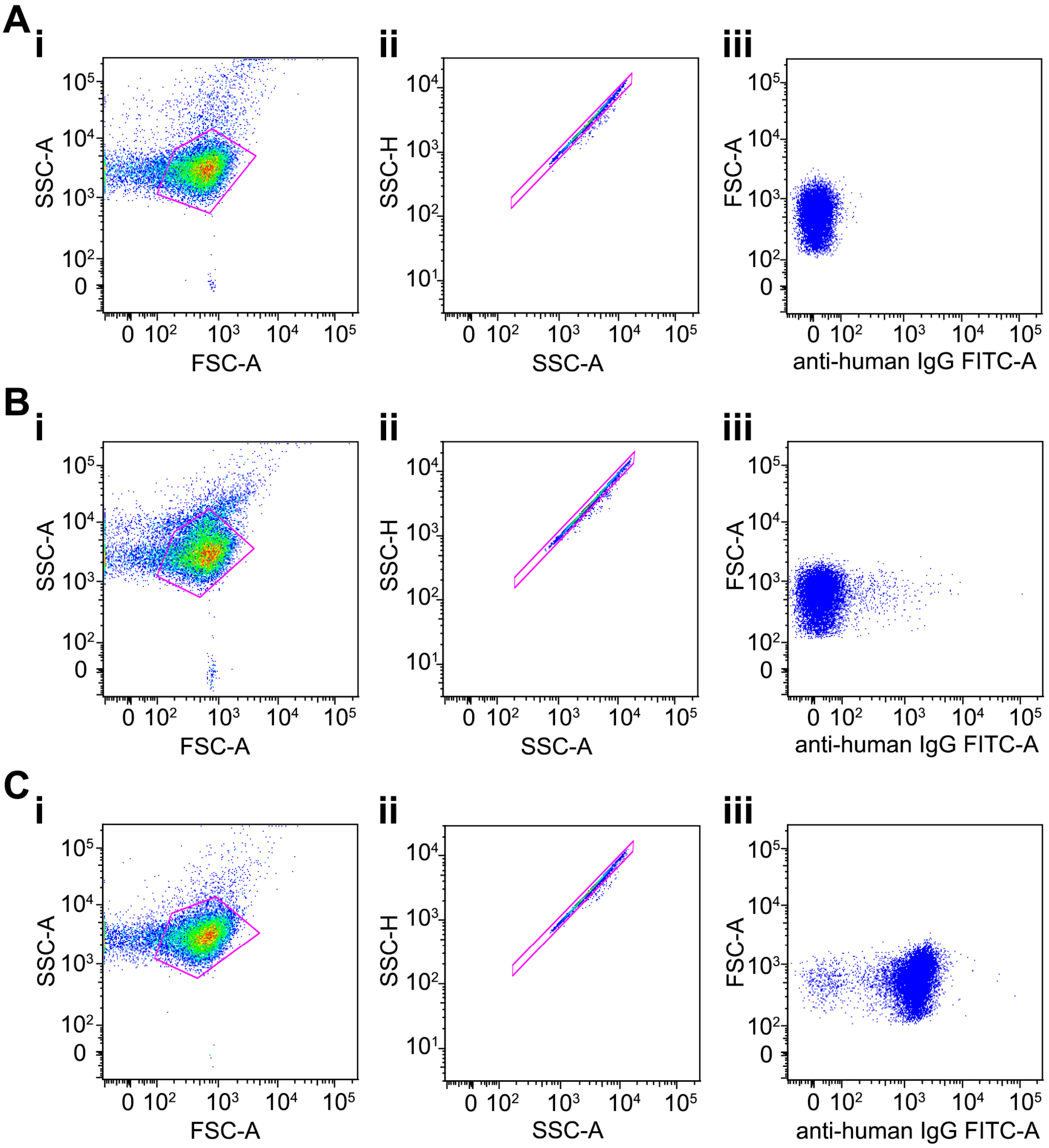

Supplementary Figure S1. Representative examples of controls used for the detection of anti-platelet antibodies by flow cytometry. Dot plots of unstained (**A**), negative (**B**) and positive (**C**) controls used to detect anti-platelet antibodies in human sera. (**Ai, Bi, Ci**) Dot plots of FSC-A versus SSC-A of all events with platelets gated. (**Aii, Bii, Cii**) Dot plots of

SSC-A versus SSC-H excluding doublets within a single cell gate. (**Aiii)** Dot plot showing no detection of anti-platelet antibodies in the unstained control. **(Biii, Ciii)** Dot plots showing detection of very low and high levels of anti-platelet antibodies in the negative **(Biii)** and positive controls (**Ciii**) respectively (generated by a secondary anti-human IgG antibody conjugated to FITC). For the unstained control, no secondary antibody was used. For the negative control, healthy human donor samples were used other than the control cohort tested to compare against stroke patients. For the positive control, a serum with known antibodies to platelet receptors was used. Positive and negative control sera were validated and supplied by the New Zealand Blood Service Centre.

Abbreviations: FSC-A, forward scatter area; IgG, immunoglobulin G; SSC-A, side scatter area; SSC-H, side scatter height.

Supplementary Figure S2.

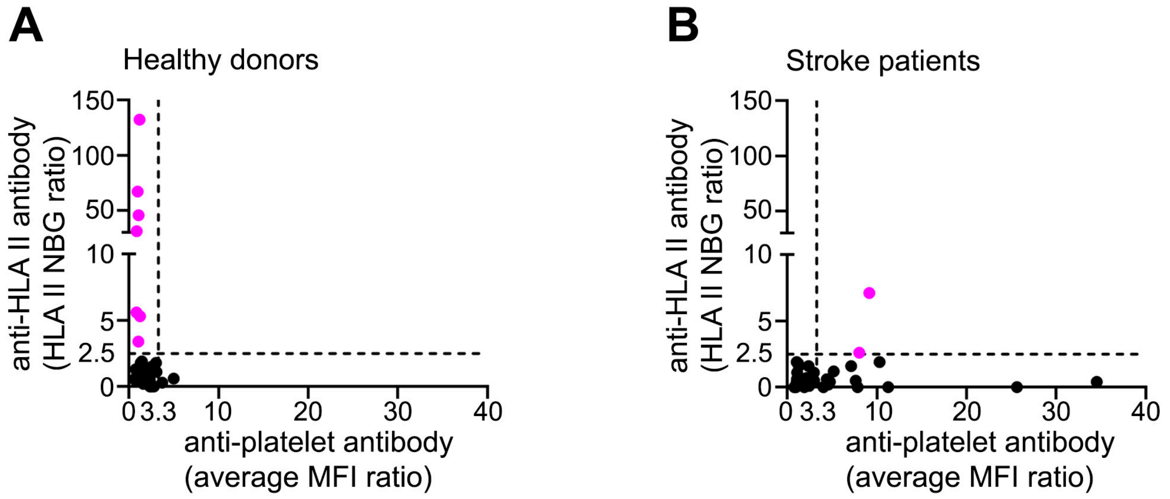

Supplementary Figure S2. Comparison between anti-platelet and anti-HLA class II antibodies in patients and controls. Relationship between anti-HLA class II antibodies and anti-platelet antibodies (average MFI ratio) in healthy donors (A**i**) and stroke patients (A**ii**). Dashed line at y = 2.5 represents the threshold for anti-HLA class II antibody positivity set as the normalised background ratio (NBG) greater than 2.5 on any of the 5 possible HLA class II beads. Dashed line at x = 3.3 represents the threshold for anti-platelet antibody positivity set based on the mean + 2 SD of healthy donors. Data points are mean of three replicates. Pink dots indicate positive samples; *n* = 48 of each group.

Abbreviations: HLA, human leukocyte antigen; NBG, normalised background ratio.

Supplementary Figure S3.

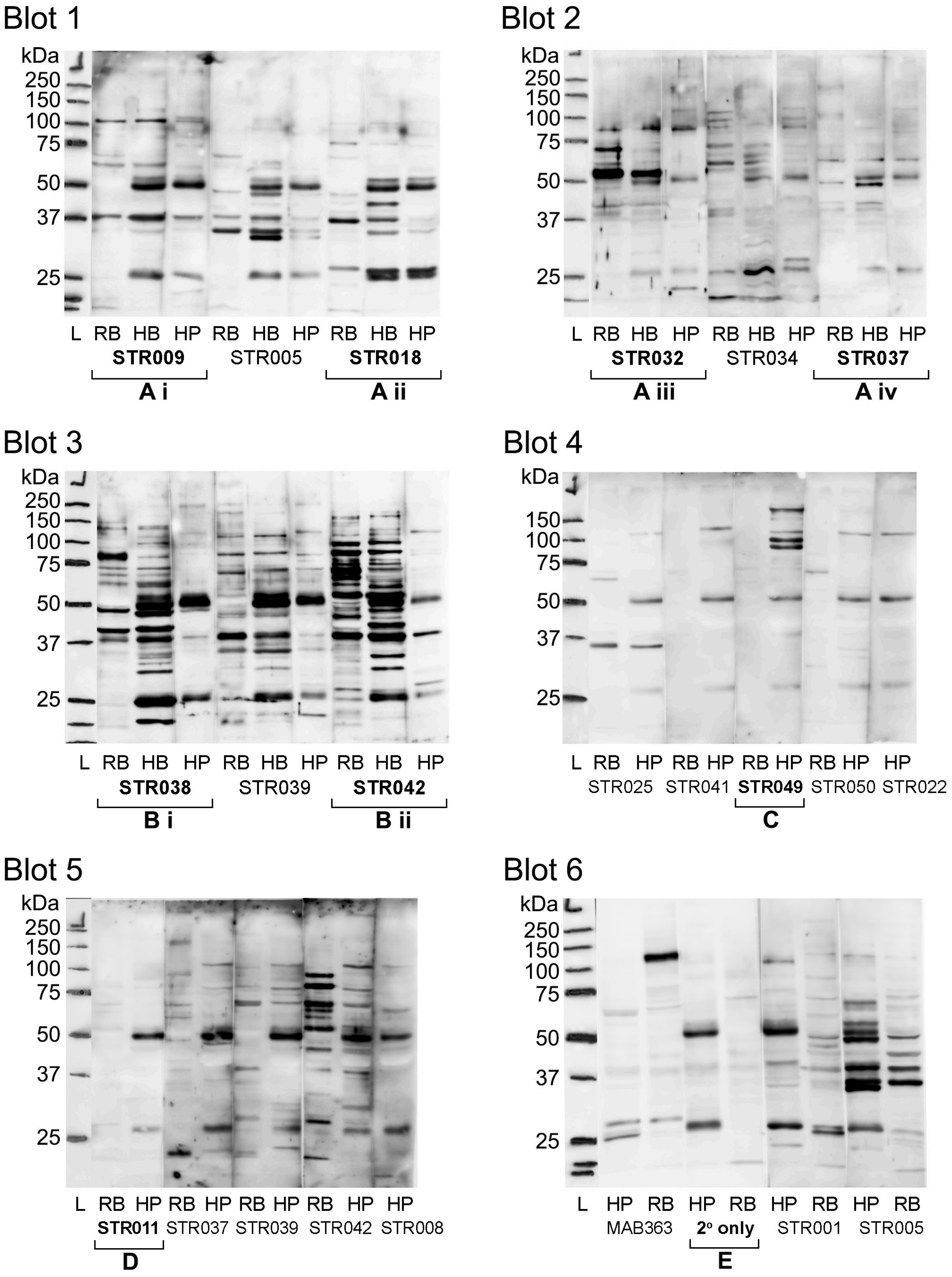

Supplementary Figure S3. Original Western blots showing binding patterns of selected stroke-derived antibodies with brain and platelet proteins. Samples used in Figure 3 are bolded and their positions in Figure 3 are indicated underneath the brackets. STR codes refer to the individual samples from stroke patients. Sera were diluted 1:500 and tested for binding to proteins extracted from rat brain tissue (RB), human brain tissue (HB) and human platelets (HP).

Supplementary Table S1: Linear regression analysis between the levels of anti-platelet antibodies and the NIHSS score, ASPECT score, and platelet counts in patients after stroke.

**Anti-platelet antibody levels**	**Linear regression**				
	**β-Coefficient Estimate**	***P*-value**	**R value**	**R^2 value**	**95% Confidence Interval**
**NIHSS score on admission (day 0)**	0.377	**< 0.001**	0.498	0.248	0.182–0.571
**NIHSS score at discharge (day 7)**	0.613	**0.001**	0.613	0.376	0.181–0.572
**ASPECT score on admission (day 0)**	-0.102	**0.003**	0.420	0.176	-0.168–(-0.037)
**Platelet counts on admission (day 0)**	13.600	**< 0.001**	0.752	0.565	8.324–18.876
**Platelet counts on discharge (day 7)**	9.100	**0.003**	0.584	0.341	3.512–14.689
